# Lattice symmetry relaxation as a cause for anisotropic line broadening and peak shift in powder diffraction

**DOI:** 10.1107/S2053273324008799

**Published:** 2024-10-03

**Authors:** Miguel Gregorkiewitz, Alice Boschetti

**Affiliations:** a Department of Physical Earth and Environmental Sciences University of Siena Italy; Institute of Crystallography - CNR, Bari, Italy

**Keywords:** Rietveld refinement, peak shapes, pseudosymmetry, microstructures

## Abstract

In powder diffraction, lattice symmetry relaxation causes a peak to split into several components which are not resolved if the degree of desymmetrization is small (pseudosymmetry). Here the equations which rule peak splitting are elaborated for the six minimal symmetry transitions, showing that the resulting split peaks are generally broader and asymmetric, and suffer an *hkl*-dependent displacement with respect to the high-symmetry parent peak. These results will be of help in Rietveld refinement of pseudosymmetric structures where an exact interpretation of peak deformation is required.

## Abstract 2

The two essential observables in powder diffraction are peak intensity and position. While the structure factor is a smooth function of the structure and changes little with desymmetrization, peak positions are more akin to delta functions and are extremely sensitive even to a small desymmetrization. It is shown that lattice relaxation causes line broadening, asymmetry and displacement which are important in the context of correct interpretation of observed powder diffraction patterns.

## 1. Introduction

In powder diffraction, peak broadening is an important issue which is increasingly used to get information about grain size and shape, microstrain and defects (Lutterotti & Scardi, 1990 ▸; Scardi *et al.*, 2018 ▸). Each of these phenomena acts in a different way on line broadening, and we distinguish between isotropic line broadening, where peak widths are a smooth function of the diffraction angle 2θ, and anisotropic broadening, where peak widths also depend either upon the orientation of the diffraction vector **Q** (typically associated with grain shape anisotropy) or upon *hkl* indices, which gives rise to fluctuations of the widths between neighbouring peaks in reciprocal space. The second case is often successfully dealt with assuming anisotropic microstrain (Popa, 1998 ▸; Stephens, 1999 ▸), but in a recent Rietveld refinement of a nanocrystalline material (Boschetti, 2013 ▸; Boschetti & Gregorkiewitz, 2023 ▸) it was found that *hkl*-dependent line broadening may also be caused by lattice symmetry relaxation which implies symmetry descent to a subgroup. In such cases, structure-independent refinement in the subgroup, using the methods of Pawley (1981 ▸) or Le Bail *et al.* (1988 ▸; see also Le Bail, 2005 ▸), is expected to improve fitting and may be used to estimate the importance of lattice relaxation. However, fitting is here to an observed powder pattern which may contain many other small peak deformations which can bias the results. Also, these techniques rely on whole powder pattern fitting which uses peak shapes (usually pseudo-Voigtian) that may not correspond at all to the actual situation in a powder pattern (Le Bail, 2000 ▸); thus, they deliver average values for unit-cell and peak shape parameters which are hard to interpret. We therefore need an alternative method to exactly control the peak deformation/displacement, due to lattice relaxation, for every single peak to be compared with observation.

Boschetti & Gregorkiewitz (2023 ▸) drew attention to (anisotropic) line broadening (ALB) in a particular case where desymmetrization from tetragonal to monoclinic was successful in explaining line widths while a microstrain model, applied without restrictions, failed to give the correct interpretation. Here we present an in-depth analysis of the effect of lattice relaxation on the peak shape in the general case of desymmetrization: it is seen that the transition to lower symmetry splits a peak into a series of components which appear, depending on the *hkl* index, at different displacements with respect to the parent high-symmetry peak, *i.e.* beyond line broadening, lattice relaxation also causes a shift of peak position and potentially creates asymmetry of the profile.

In the following, the displacements due to lattice relaxation are established for the possible six different minimal symmetry transitions, elaborating the specific formulae for each case first in terms of *Q*^2^ (in Section 2.1 and Sections 1.2 to 1.6 in the supporting information) and then in terms of 2θ (Section 3.1). Multiplied with a Voigtian, the *i* components can then be summed for comparison with the observed split peak, as will be shown below.

## 2. Possible lattice relaxations and peak splitting

Symmetry relaxations can be rationalized in the frame of group–subgroup relations where the transformation index *i* takes values of 2, 3, 4 or 6 when the transition is to a maximal subgroup. A single peak in the higher symmetry splits into ≤ *i* components in the subgroup symmetry.

Depending on the symmetry, one has to find the multiplicities of the reflections in the high- and low-symmetry case, and then establish the equations for the positions of each (component) peak. The following scheme outlines this procedure for the transition from cubic to tetragonal as an example.

The procedure to identify indices and *d* spacings of split peaks and their components is as follows:

(i) Establish the multiplicity *M* of a general reflection *hkl* in the higher unit-cell symmetry (*e.g.* cubic: *M* = 48).

(ii) Find a set of equivalent *hkl* where 1/*d*^2^ becomes different through desymmetrization (*e.g.* for cubic > tetragonal, *c* = *a* + δ*a*, we find: *hkl* − *klh* − *lhk*); this triplet is equivalent through 3_1__1__1_ in the positive octant which contains also the triplet *khl*–*lkh*–*hlk*, equivalent through 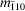 to the first. Each octant thus contains a sextuplet and we get *M* = 6 × 8 = 48.

(iii) Establish the equation 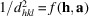 in the higher-symmetry unit cell.

(iv) Establish the equations 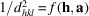 in the lower-symmetry unit cell, for the *i* (cubic > tetragonal: *i* = 3) indices *hkl* which are now non-equivalent.

(v) Get the relative position of the peaks created by desymmetrization, for the general and special reflection classes. For cubic > tetragonal, for example, we have generally three Δ(1/*d*^2^) = (1/*d*^2^)_c_ − (1/*d*^2^)_t_ values which degenerate as follows (*cf.* Fig. 2):[Chem scheme1]
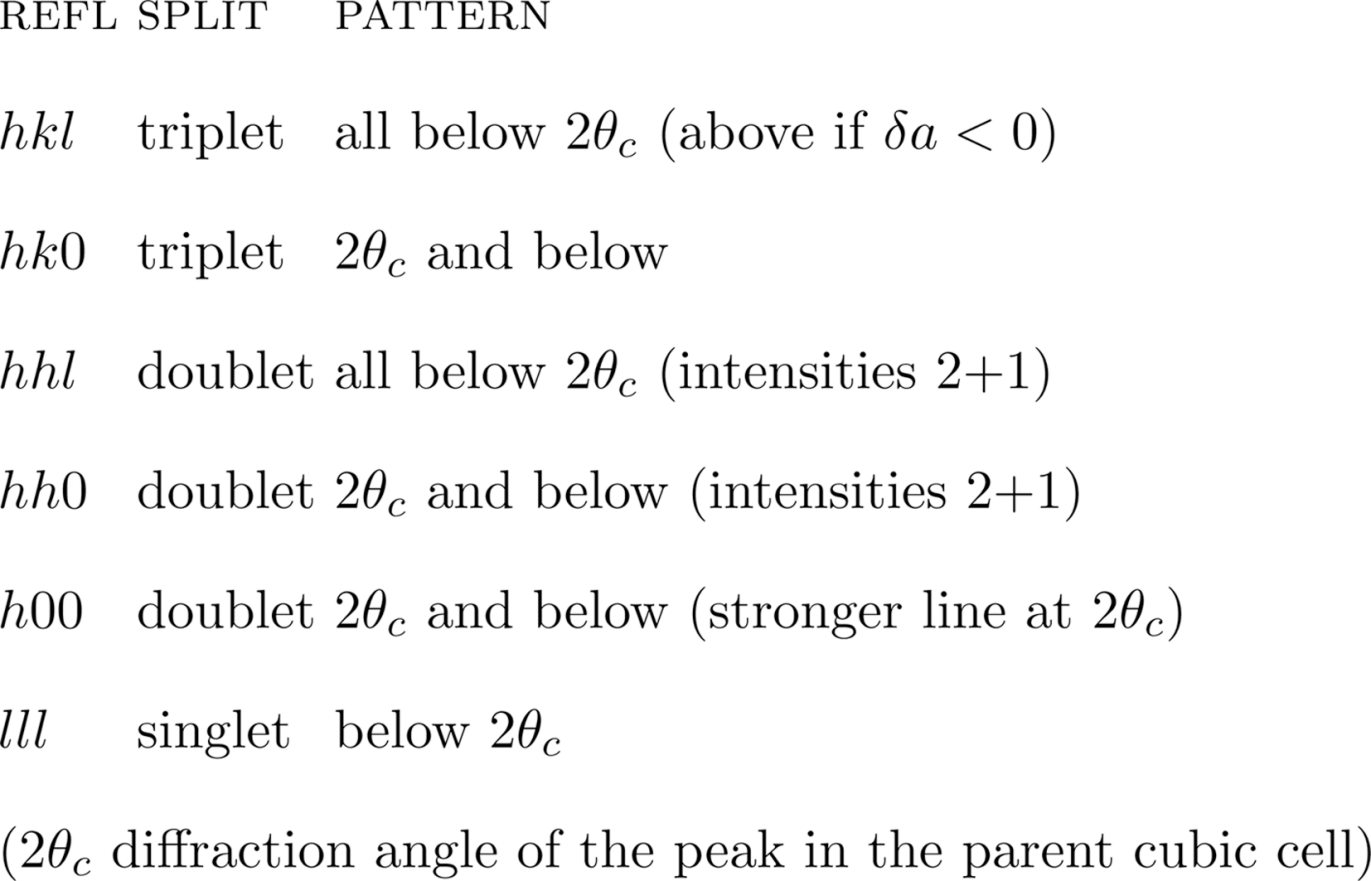


### 2.1. Cubic to tetragonal

For relaxation from cubic to tetragonal we get 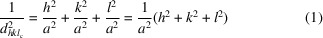

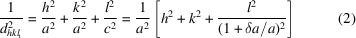

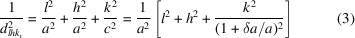

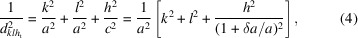
where *c* = *a* + δ*a* = *a*(1 + δ*a*/*a*) = *aD* and for δ*a* → 0, equations (2)–(4) reduce to the cubic formula in equation (1).

The relevant figures for our purpose are the positions of the split components relative to the cubic parent, *i.e.* the displacements 
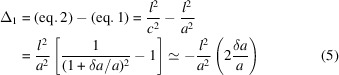

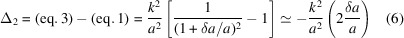

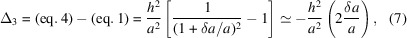
where the approximation is valid within a relative error of ε ≤ 1.5|δ*a*/*a*|, *e.g.* for |δ*a*/*a*| = 0.01(0.1) the amount of displacement |Δ| is overestimated by 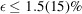.

**Figure 1:**
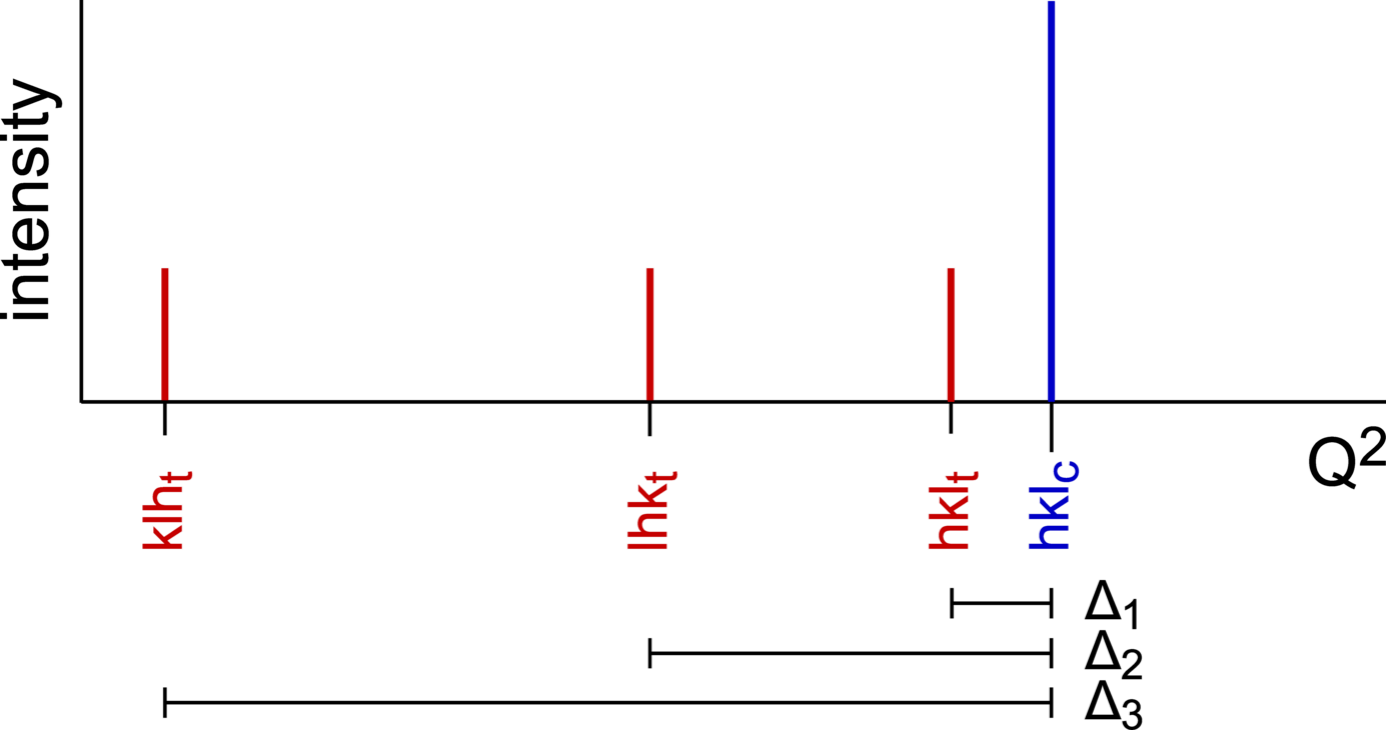
Original position (blue) of a general reflection *hkl* in the cubic parent cell and displacements Δ of the three split positions (red) after relaxation to a tetragonal cell. Relative intensities reflect the multiplicities which are *M* = 48 for the cubic and *M* = 16 for the three tetragonal positions. *hkl* = 321.

From equations (5)–(7) it is seen that Δ_*i*_ scales with the extent of desymmetrization δ*a*/*a* and the Miller index. A graphical presentation is given in Fig. 1 ▸.

Fig. 1 ▸ has been designed for a general reflection *hkl* with the values 321. In this case, the single peak in the cubic symmetry splits into *i* = 3 components in the tetragonal system and all components are displaced from the original cubic position. Several subsets of this pattern have to be considered for special reflections:[Chem scheme2]
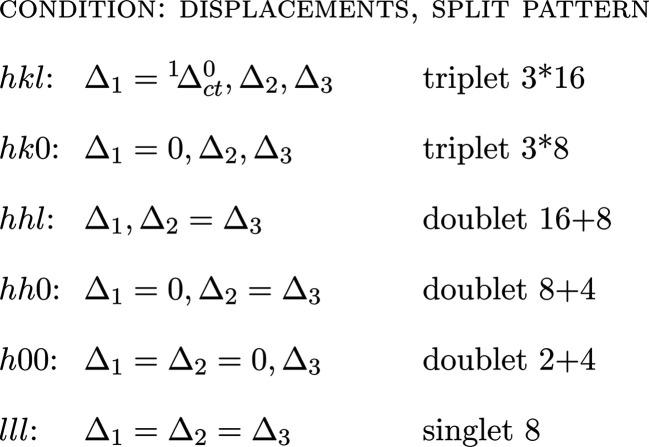


**Figure 2:**
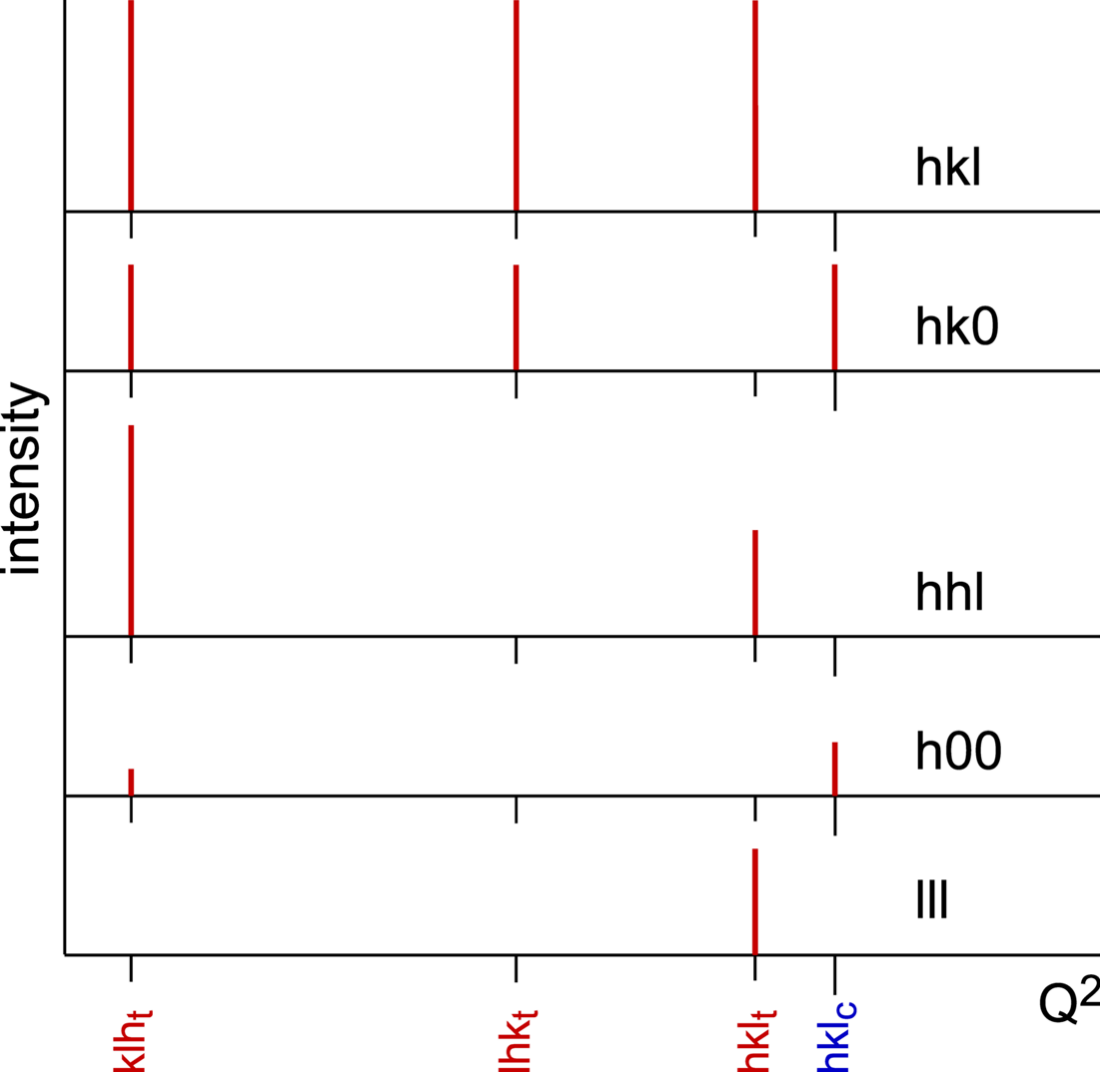
Peak splitting of general *hkl* and special reflections after relaxation from a cubic to a tetragonal unit cell. For special reflections, some displacements may become 0 or degenerate, forming doublets and singlets. The actual displacements depend on the values of *hkl* (here *hkl* = 321), but the split pattern remains the same for a given class of reflections. Intensity scale differs from Fig. 1 ▸.

Here splitting results in triplets, doublets or singlets and displacements may be nil (Δ = 0). Some of this is shown in Fig. 2 ▸.

### 2.2. Remaining five cases of lattice relaxation

The remaining five cases of lattice symmetry relaxation, *i.e*. cubic to rhombohedral, hexagonal to orthorhombic/monoclinic, tetragonal to orthorhombic, orthorhombic to monoclinic, monoclinic to anorthic (triclinic), are developed in the supporting information (Sections 1.2 to 1.6), following the same scheme as above for the cubic to tetragonal relaxation.

Some pecularities arise. In all four cases involving an angle relaxation (δγ or δα/β), there appears a barycentre slightly off the parent peak position, except for hexagonal to ortho­rhombic/monoclinic. This barycentre does not exist in the two orthogonal cases (cubic to tetragonal and tetragonal to orthorhombic). The barycentre reported in Figs. S1, S2 and S5 in the supporting information was designed for δα/γ = 1°, but the distance from the parent peak changes also with *hkl* and may increase. This becomes important for special reflection classes where a single peak may appear at the barycentre and would be simulated with the parent peak in supergroup refinement (see Appendix *A*[App appa]).

Another curiosity is the fact that in two cases involving angle relaxation, cubic to rhombohedral and hexagonal to orthorhombic/monoclinic, the displacements are not restricted to certain few values as in the other cases, and a much higher dispersion is observed (Figs. S2 and S3).

## 3. Comparison with observed peak shape and position

In Section 2 and Sections 1.2 to 1.6 in the supporting information, the displacements of the split peak components are given as line diagrams in terms of *Q*^2^, but the experimental description of a peak implies also shape and width and is usually given in terms of 2θ. The following is concerned with the calculus of the expected parameters of a non-resolvable split peak and their comparison with observation.

### 3.1. The increment in diffraction angle 2θ

From Bragg’s equation we get 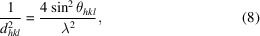
which contains the relation between the peak position in terms of *Q*^2^ and the Bragg angle θ. For the increments one obtains 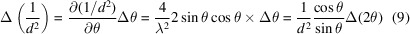
and 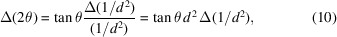
showing that the diffraction angle 2θ varies with the relative variation of *Q*^2^ and is proportional to 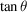, the same factor known for peak broadening due to microstrain.

Substituting *d*^2^ and Δ(1/*d*^2^) by the expressions developed above (Section 2 and Sections 1.2 to 1.6 in the supporting information) for the peak positions in the supergroup and after relaxation of the lattice symmetry, respectively, we obtain the displacements Δ_*i*_(2θ) for each of the desymmetrization schemes, now in terms of the unit-cell parameters, the reflection indices *hkl* and the relative amount of linear (δ*a*/*a*) or angular (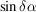) desymmetrization.

In particular, for relaxation from cubic to tetragonal, the dependence on unit cell cancels and one gets, *e.g.*, 
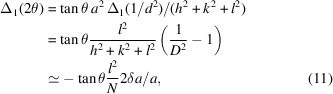
showing that Δ(2θ) scales strongly with *hkl*, and where *N* = *h*^2^ + *k*^2^ + *l*^2^ as usual. The barycentre for the three displacements is always at (*l*^2^/*N* + *k*^2^/*N* + *h*^2^/*N*)/3 = 1/3, which holds also for the singlet *lll* where the three possible displacements [equations (5)–(7)] have identical values, *i.e.* lattice relaxation may cause peak displacement alone, without an accompanying broadening.

For relaxation from cubic to rhombohedral, one finds, *e.g.*, 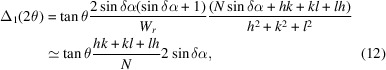
where *W_r_* = 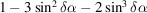 and the approximation is formally similar to the preceding case (the small summand representing the barycentre, 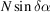, is ignored).

For the transition from hexagonal to orthorhombic, the increment in the diffraction angle is given by, *e.g.*, 
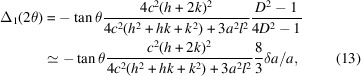
for tetragonal to orthorhombic by 
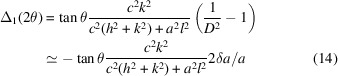
and for orthorhombic to monoclinic by 
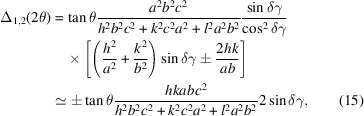
where the two possible displacements are specified.

From the last three examples it is seen that, in systems other than cubic, the scatter of split positions depends also on the unit-cell parameters which mix with the *hkl* term. One important consequence relates to the compensation of peak displacements by unit-cell refinement in the supergroup. In Appendix *A*[App appa] this issue is addressed in some depth, demonstrating that compensation will occur in most cases, but reliable results are obtained only for the desymmetrization (cubic > tetragonal) while in all other cases it delivers inadmissible results due to mixing with other cell parameters. For our purpose, this means that, in a Rietveld refinement, only peak broadening and asymmetry appear as specific for a given lattice relaxation, whereas line displacement will usually suffer from wrong refinement in the supergroup which tries to match a peak or a barycentre.

### 3.2. Generation of split peaks

Split peaks can easily be simulated by summation over single Voigtians, each centred at one of the split positions and multiplied with the corresponding multiplicities. The resulting, generally asymmetric, split peak can be compared with the calculated peak for the supergroup, in terms of position, FWHM, skewness and a difference plot of their profiles. In the following, we first show how the simulation of a split peak works assuming three different cases of FWHM (including the case of special reflection classes where no splitting occurs), and then some representative cases from the literature are discussed.

We chose here the case of desymmetrization from cubic to tetragonal which gives three split positions as defined in equations (5)–(7). To represent against 2θ, we use equation (11) which gives the three positions 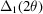 = 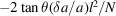, 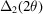 = 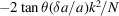 and 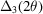 = 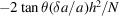 to centre the Gaussians. We chose the example of the leucite analogue KGaSi_2_O_6_ (Bell & Henderson, 2019 ▸) with *Ia*3*d* > *I*4_1_/*a*, *a*_c_ = 13.58 Å, and the reflection triplet for *hkl* = 321 as defined in Fig. 2 ▸, so that 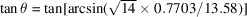 = 0.22 and Δ_1_(2θ) = −0.036°, Δ_2_(2θ) = −0.14° and Δ_3_(2θ) = −0.32°.

The values chosen for FWHM correspond to the instrumental broadening of X-rays at a synchrotron radiation source [0.008° (2θ)] (Gozzo *et al.*, 2006 ▸), the typical instrumental broadening of laboratory radiation [0.07° (2θ)] (Balzar *et al.*, 2004 ▸) and, third, a typical FWHM observed in materials with an important contribution to broadening from grain size [0.2° (2θ), corresponding to approximately 45 nm]. It is seen that the chosen reflection triplet for *hkl* = 321 in Fig. 2 ▸ is well (scarcely) resolved for synchrotron (laboratory) radiation and only a single, deformed, peak is observed for materials with small grain size. Interestingly, the simulation with the third FWHM shows clearly the problems in defining a Gaussian in the case of supergroup refinement as mentioned in Appendix *A*[App appa].

Remember that the split peak is centred at its parent peak angle, so the 2θ dependence of FWHM can be ignored. Note also that all split positions are negative, a consequence of *c*_t_ > *a*_c_ (or δ*a*/*a* > 0). For *c*_t_ < *a*_c_ (or δ*a*/*a* < 0), the displacements would appear reflected to positive angles. All simulations were performed using a home-made *scilab* (Scilab Enterprises, 2012 ▸) routine (available upon request from the authors).

**Figure 3:**
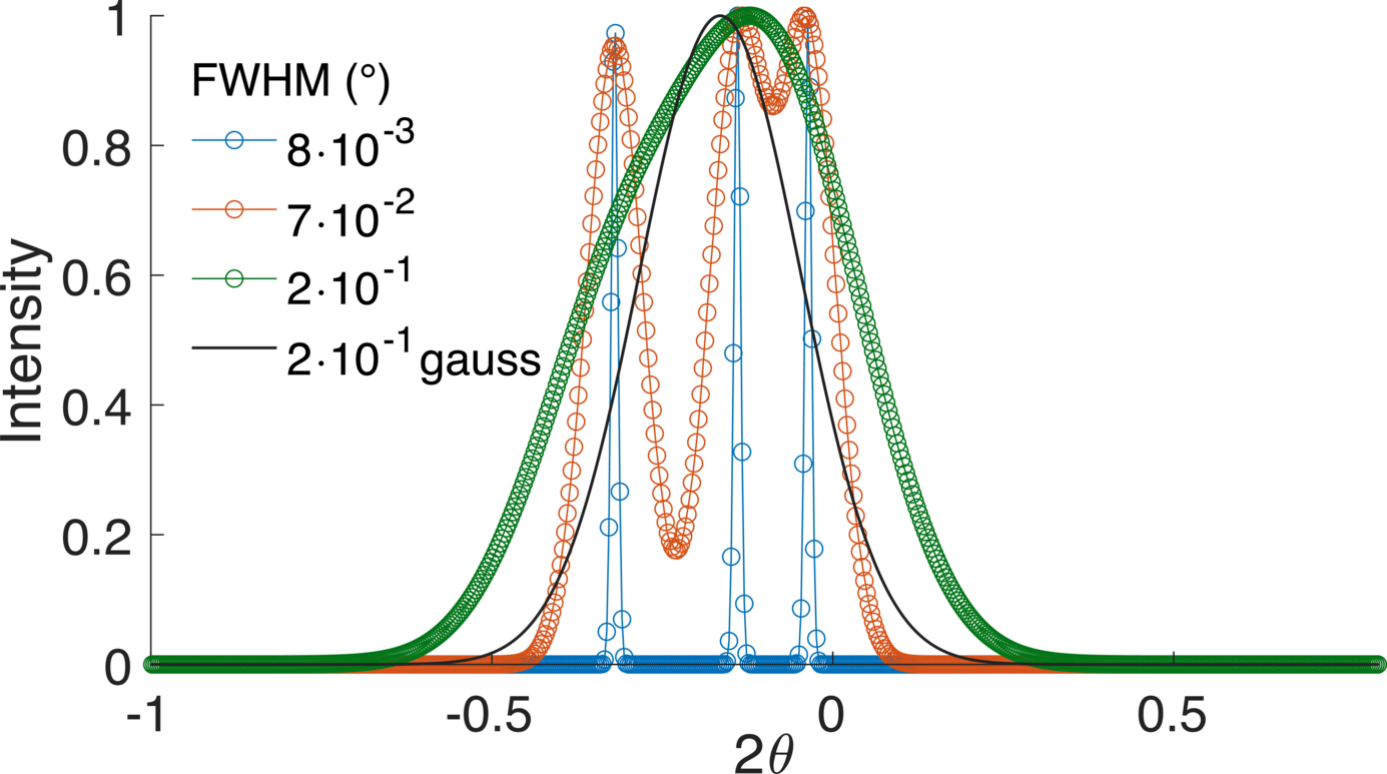
The reflection triplet for *hkl* = 321 as defined in Fig. 2 ▸, presented in a simulation of the diffraction pattern as Gaussian peaks versus 2θ, assuming three different typical FWHMs (see text).

In Fig. 3 ▸, a general *hkl* reflection and its splitting are presented, but there are cases, less common but often referring to strong reflections, of the special *hkl* reflection classes where only a single displacement exists (Fig. 2 ▸, Figs. S2–S5). In these cases, no split peak like the one in Fig. 3 ▸ is observed and lattice relaxation is expressed only by peak displacement. This may create problems with the simulation of the peak in supergroup refinement (see Appendix *A*[App appa]).

**Figure 4:**
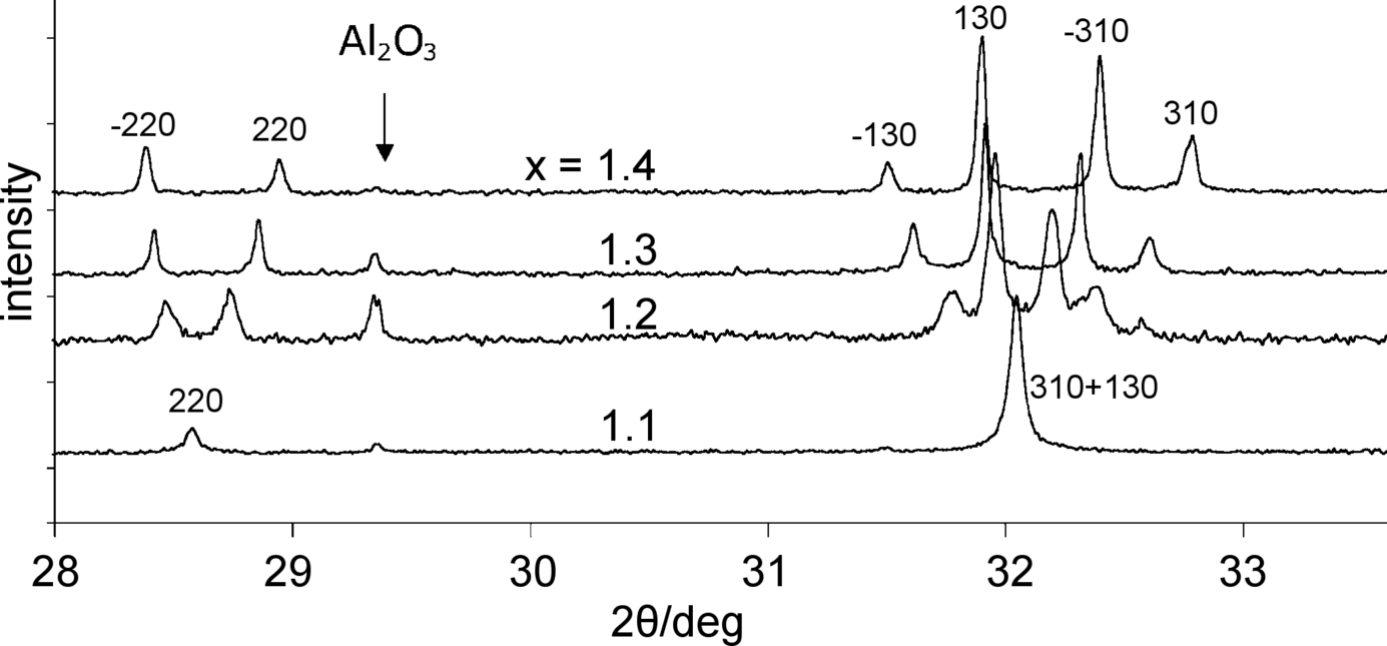
X-ray diffraction patterns of Ba_*x*_Fe_2*x*_Ti_8−2*x*_O_16_ with *x* = 1.1, 1.2, 1.3, 1.4. For *x* ≥ 1.2 the symmetry is monoclinic with increasing degree of desymmetrization. In the tetragonal case (*x* = 1.1) the reflections 310 and 130 coincide, but their intensities are different (*I*130 > *I*310) as can be seen in the cases with *x* ≥ 1.2. Modified from Carter (2004 ▸); λ = 1.7 Å, synchrotron radiation.

The first example from the literature concerns an important detail for intensities. While, in most observations, the *i* split components will have almost identical intensities, attention has to be paid in cases of merohedry in the cubic, hexagonal and tetragonal systems. A beautiful example is the transition *I*4/*m* → *I*2/*m* reported in Fig. 4 ▸ (Carter, 2004 ▸). Here, the point group 4/*m* is a merohedry, where reflections *hkl* are not equivalent to reflections *khl* as in the holohedric group 4/*m*2/*m*2/*m*, and *I*_130_ > *I*_310_ (the further splitting in *hkl* and −*hkl* is caused by the transition to monoclinic symmetry). It is evident that the width and asymmetry of the split peak depend on the ratio *I*_130_:*I*_310_. In the example of Carter (2004 ▸), the split components are well separated, but in the case of pseudosymmetric samples as considered here, the splitting would of course hardly be visible and a single broadened peak is expected, similar to the peak for *x* = 1.1 in Fig. 4 ▸.

Another problem is peak asymmetry. It has been pointed out (Leineweber, 2017 ▸; Boschetti & Gregorkiewitz, 2023 ▸) that anisotropic line broadening as found from Rietveld refinement using the model of Popa (1998 ▸), Stephens (1999 ▸), if properly applied, simulates the peak broadening due to lattice relaxation. [As an example, from Stephens (1999 ▸), the refinable anisotropy parameters in the tetragonal system are *S*_400_ = *S*_040_, *S*_220_, *S*_202_ = *S*_022_ and *S*_004_. For lattice relaxation to orthorhombic symmetry, the expected ALB is described by equations (35)–(36) in the supporting information which are invariant to index *l*, so *S*_202_ = *S*_022_ and *S*_004_ have to be set to 0.] This model can provide for anisotropic line broadening in *hkl* but there is no way to account for the peak asymmetry which is generally expected for lattice relaxation (see Figs. 1 ▸, S1, S3). To compare the asymmetry of the peak expected for lattice relaxation, the observed peak’s asymmetry can be assessed by single peak refinement allowing for skewness.

## 4. Experimental evidence for lattice symmetry relaxation

Lattice relaxation as a cause of ALB was first discussed (Boschetti & Gregorkiewitz, 2023 ▸) in the Rietveld refinement of cryptomelane K_*x*_[Mn_8_O_16_] nanocrystals, where a small size is desirable for application as electrode or supercapacitor material. Observed line widths were in the order of 0.5–1.5° (2θ) and the anisotropic fluctuations could consistently be explained assuming needle-like morphology and lattice relaxation, the latter being essential to obtain an unbiased particle shape of 61 × 61 × 178 Å^3^. Refinement in the monoclinic subgroup *I*112/*m* was attempted [see the supporting information of Boschetti & Gregorkiewitz (2023 ▸)] but, with such line widths, peak overlap at higher angles 2θ becomes prohibitive and no better result could be obtained.

A literature search on cryptomelane Rietveld refinements revealed that there are many cases where a tetragonal *I*4/*m* symmetry was used (*e.g.* Gao *et al.*, 2008 ▸; Huang *et al.*, 2018 ▸; Chong *et al.*, 2018 ▸), and inspection of the difference intensity plots reveals that correction for shape anisotropy has been applied in the two former examples, whereas anisotropic microstrain (Stephens, 1999 ▸) or lattice relaxation, though visibly present, have apparently not been considered. In one case (Espinal *et al.*, 2012 ▸), with considerably smaller line widths, Rietveld refinement was conducted in *I*2/*m*, but ALB was not discussed and a difference profile has not been published.

A clear case of lattice relaxation has been reported for pyrolusite MnO_2_ (Fabrykiewicz *et al.*, 2019 ▸). This structure is normally described in space group *P*4_2_/*mnm* but line broadening is strongly anisotropic and has successfully been modelled either using the Stephens (1999 ▸) microstrain model in the tetragonal supergroup or passing to the orthorhombic subgroup *Pnnm*. Interestingly, the higher-order reflection 400 in the orthorhombic model is well separated from other peaks due to a much smaller unit cell than in cryptomelane and, with a somewhat smaller peak width, a feeble splitting of this peak is expected but not observed. An excellent fit could however be achieved assuming a Gaussian distribution of very slightly different orthorhombic unit cells, with *a*/*b* ranging from 4.39 to 4.40 Å.

While refinement in the subgroup is certainly preferable, we have seen that this alternative to a description using anisotropic line broadening is often hampered by peak overlap at higher angles which increases with the dimensions and lower symmetry of the unit cell. In fact, even with peak widths of 0.05° and less it may occur that the first, isolated, peaks are not resolved while peaks at higher angles are strongly overlapping. A nice example of this situation is the structure of MFI, heavily studied for zeolite membranes (Weng *et al.*, 2023 ▸), where a large unit cell undergoes transition from ortho­rhombic (Kokotailo *et al.*, 1978 ▸) to monoclinic (Wu *et al.*, 1979 ▸). Here, reflection density for the monoclinic cell is so high that sometimes an orthorhombic cell is alternatively used (Leardini *et al.*, 2014 ▸). In principle, there is no limit on line widths and, even with synchrotron radiation, refinement in the subgroup symmetry might run into difficulties.

From a practical point of view, incorporation of lattice relaxation in a Rietveld code would be most easy using a separation between the symmetries used for the crystal structure (which could remain the supergroup) and the unit cell (which lowers to the subgroup). In this way, atom parameters can be refined without correlations, and the unit cell can adapt for lattice relaxation. It is interesting that a separation between these symmetries has recently been developed (Perez-Mato *et al.*, 2010 ▸; Kerman *et al.*, 2012 ▸) and successfully used (Lewis *et al.*, 2016 ▸; Rousse *et al.*, 2017 ▸) in symmetry mode triggered Rietveld methods where a restrained structural refinement is undertaken in the frame of the subgroup unit cell.

In desymmetrization, a separation between the atom and the unit-cell parameters complies with a fundamental observation: while the structure factor is a smooth function of atom parameters and varies little with desymmetrization, peak positions are more akin to delta functions which vary dramatically, even with a small desymmetrization of the unit cell. Lattice relaxation is therefore of fundamental importance for Rietveld refinement in pseudosymmetric cases.

## 5. Conclusions

The present findings can be applied to interpret peak misfits in Rietveld refinement of pseudosymmetric materials. Misfit may arise from line displacement and/or broadening due to lattice symmetry relaxation. Both phenomena scale with the amount of desymmetrization δ*a*/*a*, δα and depend on the reflection index *hkl*, with typical split patterns for different reflection classes. Often, a single desymmetrization parameter gives rise to a wealth of splitting schemes (and resulting peak shapes and displacements) which would be difficult to simulate otherwise.

In the future, lattice symmetry relaxation might be adopted as a routine in Rietveld code; this becomes particularly important if other causes of ALB, such as grain size and microstrain anisotropy, have to be refined to a meaningful microstructure model.

## Supplementary Material

Supporting information. DOI: 10.1107/S2053273324008799/ae5149sup1.pdf

## Appendix A Compensation of line displacement in supergroup refinement

In Section 3.1 it was stated that line displacement may to some extent be compensated in supergroup refinement. In the following, three different cases of desymmetrization are discussed to show the intricacies of such compensation.

In the first case (cubic > tetragonal), the first displacement is given by equation (11), and from the sum of the three possible displacements one gets the shift of barycentre,

due to desymmetrization, that can be compared with the centre of the line obtained from refinement in the (cubic) supergroup which, applying equation (10), is

where *D*′^2^ = 3*D*^2^/(1 + 2*D*^2^) ≃ 1 + (*D*^2^ − 1)/3, *i.e.* the mantissa of *D*′^2^ is about 1/3 that of *D*^2^, as expected. There is, however, a conceptual difference between the two cases: while equation (16) refers to the barycentre of a generally asymmetric split peak, equation (17) refers to a symmetric peak which may be simulated with various degrees of broadening but will always remain a single line, centred at the maximum of intensity. This difference is expected to be small, so we can say that cell enlargement in the supergroup refinement reflects reasonably well the line displacement due to desymmetrization.

In the second case (cubic > rhombohedral), the first displacement is given by equation (12), and from the sum of the four possible displacements one gets the barycentre,

The displacement of the line in the (cubic) supergroup refinement is

*i.e.* the line displacement cannot be simulated in the supergroup where α is constrained to 90°. The displacement of the barycentre calculated from equation (18) is minimal anyway, with  for δα = 0.95° which is about 1% of the displacement due to δ*a*/*a* = 0.025, two typical values of desymmetrization taken from the cryptomelane structure cited by Post *et al.* (1982 ▸).

In the third example for desymmetrization (tetragonal > orthorhombic), the first of two possible displacements is given by equation (14) and the barycentre is at

which is compared with the line shift in the (tetragonal) supergroup refinement,

where an increment in the *c* axis, *D*_*c*_, not foreseen by desymmetrization, appears together with *D*_*a*_ in a complicated way. A compensation of line displacement in this case is obviously possible but involves the *c* axis which should stay independent, as seen in equation (20).

Concluding, one finds that a virtually true compensation is possible for the case cubic > tetragonal, whereas it is impossible for desymmetrization (cubic > rhombohedral) and renders inadmissible results in all other cases due to mixing with other unit-cell parameters which are actually not affected by desymmetrization.
