# Supplementary material for: Lattice symmetry relaxation as a cause for anisotropic line broadening and peak shift in powder diffraction
Source: Acta Crystallogr A Found Adv. 2024 Oct 3;80(Pt 6):439–45. doi: 10.1107/S2053273324008799 (PMC11532927; doi:10.1107/S2053273324008799)
Supplement: Supplementary file 1 [file a-80-00439-sup1.pdf]

Acta Cryst A FOUNDATIONS ADVANCES

Volume 80 (2024)

Supporting information for article:

Lattice symmetry relaxation as a cause for anisotropic line broadening and peak shift in powder diffraction

Miguel Gregorkiewitz and Alice Boschetti

Supporting Information to: Lattice symmetry relaxation as a cause for anisotropic line broadening and peak shift in powder diffraction

Miguel Gregorkiewitz* and Alice Boschetti[1]

Department of Physical, Earth and Environmental Sciences, University of Siena, Italy. E-mail: gregormigu@gmail.com,a.boschetti@inrim.it

1. Possible lattice relaxations and peak splitting

The case for the transition from cubic to tetragonal has been developed in the main paper, reporting the formulae for the absolute and relative displacements of the diffraction peaks, with two figures containing the supergroup and subgroup reflection positions, and a scheme for the subgroup reflection positions in special *hkl* groups.

Here, in the Supporting Information, after the title for the cubic to tetragonal transition, the development for the remaining five lattice relaxations is reported: cubic to rhombohedral, hexagonal to orthorhombic/monoclinic, tetragonal to orthorhombic, orthorhombic to monoclinic, monoclinic to anorthic (triclinic).

1.1. Cubic to tetragonal

This case has been treated in the main paper (section 2.1).

[1] Present address: National Institute of Metrology (INRIM), Strada delle Cacce 91, I-10135 Turin, Italy; European Laboratory for Non-Linear Spectroscopy (LENS), University of Florence, via Nello Carrara 1, I-50019 Sesto Fiorentino, Italy.

1.2. Cubic to rhombohedral

For desymmetrization from cubic to rhombohedral we write

$$\frac{1}{d^2_{hkl_c}} = \frac{h^2}{a^2} + \frac{k^2}{a^2} + \frac{l^2}{a^2} = \frac{1}{a^2 W_r}(h^2 + k^2 + l^2)W_r \quad (1)$$

$$\frac{1}{d^2_{hkl_r}} = \frac{1}{a^2 W_r}[(h^2 + k^2 + l^2)\cos^2\delta\alpha + \\ + (hk + kl + lh)2\sin\delta\alpha(1 + \sin\delta\alpha)] \quad (2)$$

$$\frac{1}{d^2_{hk\bar{l}_r}} = \frac{1}{a^2 W_r}[(h^2 + k^2 + l^2)\cos^2\delta\alpha + \\ + (hk - kl - lh)2\sin\delta\alpha(1 + \sin\delta\alpha)] \quad (3)$$

$$\frac{1}{d^2_{h\bar{k}l_r}} = \frac{1}{a^2 W_r}[(h^2 + k^2 + l^2)\cos^2\delta\alpha + \\ + (-hk - kl + lh)2\sin\delta\alpha(1 + \sin\delta\alpha)] \quad (4)$$

$$\frac{1}{d^2_{\bar{h}kl_r}} = \frac{1}{a^2 W_r}[(h^2 + k^2 + l^2)\cos^2\delta\alpha + \\ + (-hk + kl - lh)2\sin\delta\alpha(1 + \sin\delta\alpha] \quad (5)$$

where $\alpha = (90 + \delta\alpha)^{\mathrm{o}}$ and $W_r = 1 - 3\sin^2\delta\alpha - 2\sin^3\delta\alpha \to 1$ and use was made of the trigonometric addition theorems in

$$\sin^2\alpha = \sin^2(90 + \delta\alpha) = \cos^2(\delta\alpha) \quad (6)$$

and

$$\cos\alpha = \cos(90 + \delta\alpha) = -\sin(\delta\alpha). \quad (7)$$

Splitting generates now, for the general hkl reflection, $i = 4$ positions and their

displacements from the cubic parent are

$$\Delta_1 = (2) - (1) = \frac{1}{a^2 W_r}[(h^2+k^2+l^2)(\cos^2\delta\alpha \ - W_r) +$$
$$+ (hk+kl+lh)2\sin\delta\alpha(1+\sin\delta\alpha)] =$$
$$= \frac{2\sin\delta\alpha(1+\sin\delta\alpha)}{a^2 W_r}[(h^2+k^2+l^2)\sin\delta\alpha + (hk+kl+lh)]$$
$$\approx \frac{hk+kl+lh}{a^2} 2\sin\delta\alpha \quad (8)$$

$$\Delta_2 = (3) - (1) = \frac{2\sin\delta\alpha(1+\sin\delta\alpha)}{a^2 W_r}[(h^2+k^2+l^2)*$$
$$* \sin\delta\alpha + (hk-kl-lh)] \approx \frac{hk-kl-lh}{a^2} 2\sin\delta\alpha \quad (9)$$

$$\Delta_3 = (4) - (1) = \frac{2\sin\delta\alpha(1+\sin\delta\alpha)}{a^2 W_r}[(h^2+k^2+l^2)*$$
$$* \sin\delta\alpha + (-hk-kl+lh)] \approx \frac{-hk-kl+lh}{a^2} 2\sin\delta\alpha \quad (10)$$

$$\Delta_4 = (5) - (1) = \frac{2\sin\delta\alpha(1+\sin\delta\alpha)}{a^2 W_r}[(h^2+k^2+l^2)*$$
$$* \sin\delta\alpha + (-hk+kl-lh)] \approx \frac{-hk+kl-lh}{a^2} 2\sin\delta\alpha. \quad (11)$$

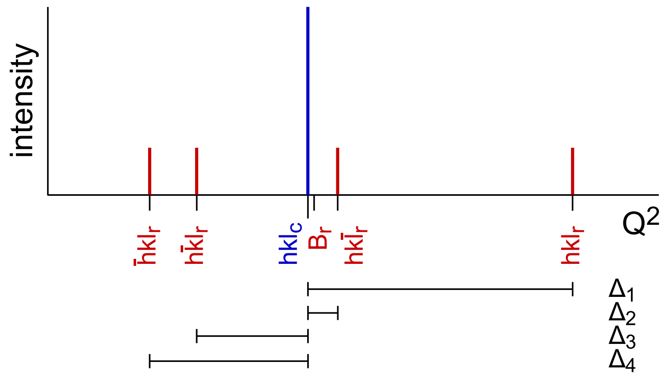

Fig. S1. Original position (blue) of a general reflection hkl in the cubic parent cell and displacements Δ of the four split positions (red) after relaxation to a rhombohedral cell. Relative intensities reflect the multiplicities ($M = 48$ and $M = 12$), $hkl = 321$ as above.

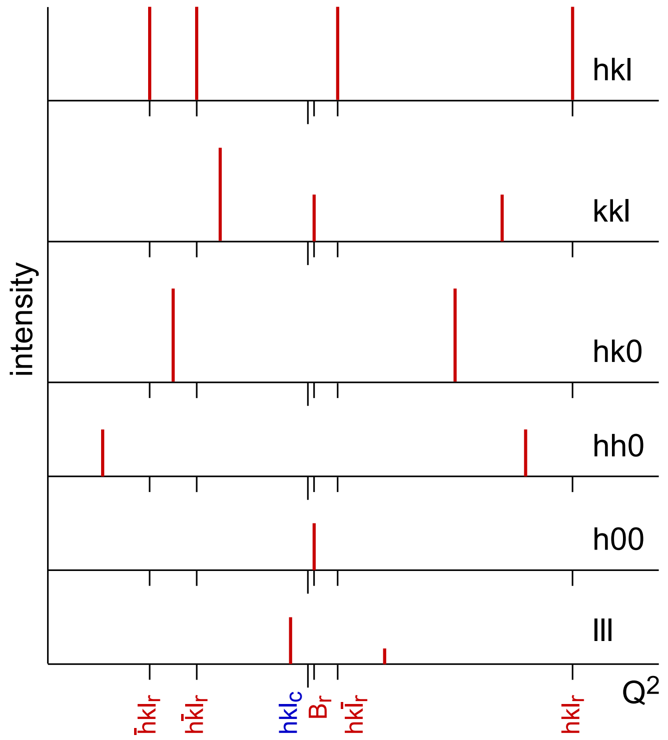

Fig. S2. Peak splitting of general hkl and special reflections after unit cell symmetry relaxation from cubic to rhombohedral. For special reflections, some displacements may become B_r or degenerate forming triplets, doublets and singlets. Actual displacements depend on the values of hkl (here $hkl = 321$), but the split pattern remains the same for a given class of reflections. The small distance between hkl_c and the barycentre B_r corresponds to $\delta\alpha = 1^{\rm o}$ and changes with hkl.

For the arbitrarily chosen general reflection 321, we now get one strongly positive peak shift (Δ_1), one peak slightly above the barycentre $B_r = (h^2 + k^2 + l^2)\sin\delta\alpha$, and two peaks below the barycentre (Fig. S1), and the conditions for special reflections are

CONDITION: DISPLACEMENTS, SPLIT PATTERN

hkl:	$\Delta_1, \Delta_2, \Delta_3, \Delta_4$	quadruplet 4*12
kkl:	$\Delta_1, \Delta_2, \Delta_3 = \Delta_4$	triplet 12+6+6
$hk0$:	$\Delta_1 = \Delta_2, \Delta_3 = \Delta_4$	doublet 12+12
$hh0$:	$\Delta_1 = \Delta_2, \Delta_3 = \Delta_4$	doublet 6+6

$h00$: $\Delta_1 = \Delta_2 = \Delta_3 = \Delta_4 = B_r$ singlet 6

lll: $\Delta_1, \Delta_2 = \Delta_3 = \Delta_4$ doublet 6+2.

For $|\delta\alpha| \leq 1^{\circ}$, the approximation in equations (8) - (11) underestimates $|\Delta|$ by the relative error $\epsilon \approx 2|\sin\delta\alpha|$ (for $\delta\alpha = 1^{\circ}$, *e.g.*, $\epsilon \approx 4\%$), but ϵ depends on the values of hkl and may be doubled if the indices are very dissimilar.

1.3. Hexagonal to orthorhombic/monoclinic

The hexagonal and trigonal crystal systems have the same (hexagonal) unit cell symmetry and the possible desymmetrizations to a maximal subgroup are either hexagonal-orthorhombic (multiplicity 24-8) or trigonal-monoclinic (multiplicity 24-4). We therefore choose to treat the two cases together in a hexagonal-monoclinic frame (multiplicity 24-4) where the orthorhombic intermediate can be considered as a special condition.

In this way, a primitive cell can be used throughout and the condition for an orthorhombic unit cell is expressed by the relation

$$\cos\gamma = -\frac{a}{2b} = -\frac{1}{2(1+\delta a/a)} = -\frac{1}{2D} \tag{12}$$

where the deviation from hexagonal $\gamma = 120^{\circ}$ is negative ($\delta\gamma \leq 0$) if $\delta a \geq 0$. The corresponding orthorhombic (desymmetrized “orthohexagonal”) cell can be obtained by transformation $\mathbf{a_o} = \mathbf{a}, \mathbf{b_o} = \mathbf{a} + \mathbf{2b}$. Note also that the “first” setting with unique axis $\mathbf{c}$ is used for the monoclinic cell to keep consistency with the hexagonal system.

The positions of a general reflection in the hexagonal cell and its six split components after desymmetrization to monoclinic are then given by

$$\frac{1}{d^2_{hkl_h}} = \frac{4(h^2+hk+k^2)}{3a^2} + \frac{l^2}{c^2} = \frac{1}{a^2}(h^2\frac{4}{3} + k^2\frac{4}{3} + hk\frac{4}{3}) + \frac{l^2}{c^2} \tag{13}$$

$$\frac{1}{d^2_{hkl_m}} = [\frac{h^2}{a^2} + \frac{k^2}{b^2} - \frac{2hk}{ab}\cos\gamma]/\sin^2\gamma + \frac{l^2}{c^2} =$$
$$= \frac{1}{a^2}[h^2\frac{1}{S} + k^2\frac{1}{SD^2} + hk\frac{C}{SD}] + \frac{l^2}{c^2} \quad (14)$$

$$\frac{1}{d^2_{khl_m}} = \frac{1}{a^2}[k^2\frac{1}{S} + h^2\frac{1}{SD^2} + hk\frac{C}{SD}] + \frac{l^2}{c^2} \quad (15)$$

$$\frac{1}{d^2_{\bar{k},h+k,l_m}} = \frac{1}{a^2}[k^2\frac{1}{S} + (h+k)^2\frac{1}{SD^2} + \bar{k}(h+k)\frac{C}{SD}] + \frac{l^2}{c^2} =$$
$$= \frac{1}{a^2}[k^2(\frac{1}{S} + \frac{1}{SD^2} - \frac{C}{SD}) + h^2\frac{1}{SD^2} + hk(\frac{2}{SD^2} - \frac{C}{SD})] + \frac{l^2}{c^2} \quad (16)$$

$$\frac{1}{d^2_{\bar{h},h+k,l_m}} = \frac{1}{a^2}[h^2(\frac{1}{S} + \frac{1}{SD^2} - \frac{C}{SD}) + k^2\frac{1}{SD^2} +$$
$$+ hk(\frac{2}{SD^2} - \frac{C}{SD})] + \frac{l^2}{c^2} \quad (17)$$

$$\frac{1}{d^2_{\bar{h}+\bar{k},h,l_m}} = \frac{1}{a^2}[h^2(\frac{1}{S} + \frac{1}{SD^2} - \frac{C}{SD}) + k^2\frac{1}{S} + hk(\frac{2}{S} - \frac{C}{SD})] + \frac{l^2}{c^2} \quad (18)$$

$$\frac{1}{d^2_{\bar{h}+\bar{k},k,l_m}} = \frac{1}{a^2}[k^2(\frac{1}{S} + \frac{1}{SD^2} - \frac{C}{SD}) + h^2\frac{1}{S} + hk(\frac{2}{S} - \frac{C}{SD})] + \frac{l^2}{c^2} \quad (19)$$

where

$$D = \frac{b}{a} = 1 + \delta a/a \rightarrow 1 \quad (20)$$

$$C = -2\cos\gamma = -2\cos(120 + \delta\gamma) =$$
$$= \cos\delta\gamma + \sqrt{3}\sin\delta\gamma \approx 1 + \sqrt{3}\sin\delta\gamma \rightarrow 1 \quad (21)$$

$$S = \sin^2\gamma = \sin^2(120 + \delta\gamma) =$$
$$\frac{3}{4} - \frac{1}{2}\sin^2\delta\gamma - \frac{\sqrt{3}}{4}\sin 2\delta\gamma \approx \frac{3}{4} - \frac{\sqrt{3}}{2}\sin\delta\gamma \rightarrow \frac{3}{4} \quad (22)$$

are parameters containing the desymmetrizations $b = a + \delta a$ and $\gamma = 120 + \delta\gamma$. For zero desymmetrization they adopt special values and equations (14) - (19) reduce to the ideal hexagonal case in equation (13).

The relative displacements are given by

$$\Delta_1 = (14) - (13) = \frac{1}{a^2}[h^2(\frac{1}{S} - \frac{4}{3}) + k^2(\frac{1}{SD^2} - \frac{4}{3}) + hk(\frac{C}{SD} - \frac{4}{3})] \quad (23)$$

$$\Delta_2 = (15) - (13) = \frac{1}{a^2}[k^2(\frac{1}{S} - \frac{4}{3}) + h^2(\frac{1}{SD^2} - \frac{4}{3}) + hk(\frac{C}{SD} - \frac{4}{3})] \quad (24)$$

$$\Delta_3 = (16) - (13) = \frac{1}{a^2}[k^2(\frac{1}{S} + \frac{1}{SD^2} - \frac{C}{SD} - \frac{4}{3}) + h^2(\frac{1}{SD^2} - \frac{4}{3}) + hk(\frac{2}{SD^2} - \frac{C}{SD} - \frac{4}{3})] \quad (25)$$

$$\Delta_4 = (17) - (13) = \frac{1}{a^2}[h^2(\frac{1}{S} + \frac{1}{SD^2} - \frac{C}{SD} - \frac{4}{3}) + k^2(\frac{1}{SD^2} - \frac{4}{3}) + hk(\frac{2}{SD^2} - \frac{C}{SD} - \frac{4}{3})] \quad (26)$$

$$\Delta_5 = (18) - (13) = \frac{1}{a^2}[h^2(\frac{1}{S} + \frac{1}{SD^2} - \frac{C}{SD} - \frac{4}{3}) + k^2(\frac{1}{S} - \frac{4}{3}) + hk(\frac{2}{S} - \frac{C}{SD} - \frac{4}{3})] \quad (27)$$

$$\Delta_6 = (19) - (13) = \frac{1}{a^2}[k^2(\frac{1}{S} + \frac{1}{SD^2} - \frac{C}{SD} - \frac{4}{3}) + h^2(\frac{1}{S} - \frac{4}{3}) + hk(\frac{2}{S} - \frac{C}{SD} - \frac{4}{3})] \quad (28)$$

which degenerate, after introducing the orthorhombicity condition (12) in equations (21) and (22) giving $C = 1/D$ and $S = 1 - 1/4D^2$, to three simpler expressions

$$\Delta_1 = \Delta_4 = -\frac{1}{a^2}\frac{4}{3}[h^2 + 4k^2 + 4hk]\frac{D^2 - 1}{4D^2 - 1} \approx -\frac{1}{a^2}[(h + 2k)^2]\frac{8}{9}\delta a/a \quad (29)$$

$$\Delta_2 = \Delta_3 = -\frac{1}{a^2}\frac{4}{3}[k^2 + 4h^2 + 4hk]\frac{D^2 - 1}{4D^2 - 1} \approx -\frac{1}{a^2}[(k + 2h)^2]\frac{8}{9}\delta a/a \quad (30)$$

$$\Delta_5 = \Delta_6 = -\frac{1}{a^2}\frac{4}{3}[h^2 + k^2 - 2hk]\frac{D^2 - 1}{4D^2 - 1}$$
$$\approx -\frac{1}{a^2}[(h-k)^2]\frac{8}{9}\delta a/a \quad (31)$$

that apply for desymmetrization of the hexagonal but not the trigonal crystal system, and where the approximation holds within a relative error (overestimation) of $\epsilon = 2.2\delta a/a$.

Special reflection classes are given below for the cases of relaxation from hexagonal/trigonal to monoclinic and hexagonal to orthorhombic.

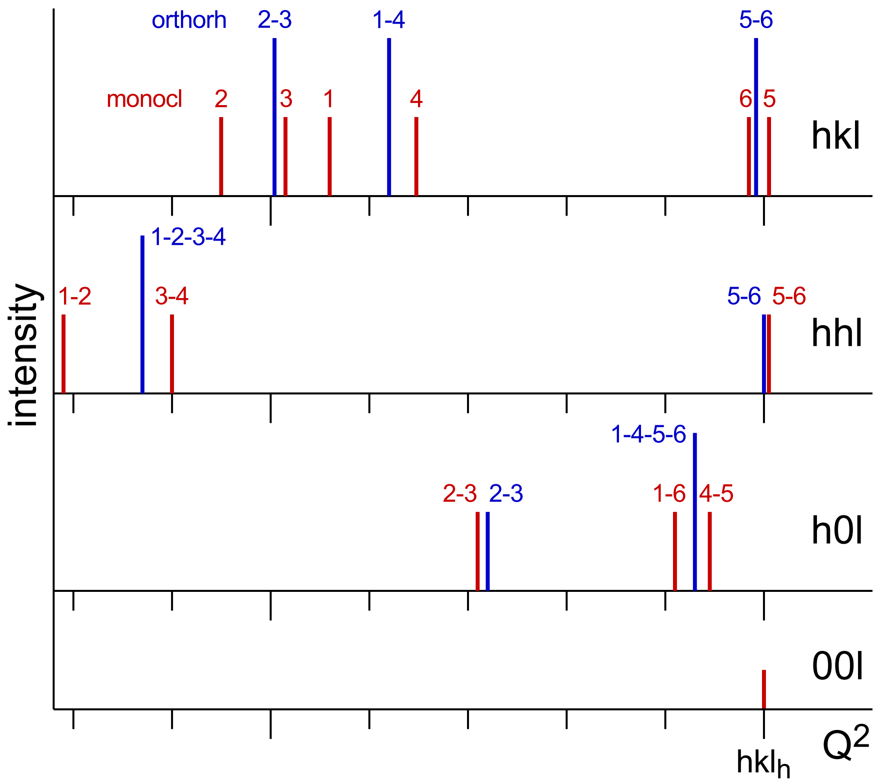

Fig. S3. Peak splitting of general hkl and special reflections after unit cell symmetry relaxation from hexagonal to orthorhombic (blue) and monoclinic (red). For special reflections, some displacements may become zero or degenerate forming triplets, doublets and singlets. Actual displacements depend on the values of hkl (here $hkl = 321$), but the split pattern remains the same for a given class of reflections. Numbers refer to Δ as defined in equations (23) - (31).

hexagonal > monoclinic ($i = 6$)

hkl: $\Delta_1, \Delta_2, \Delta_3, \Delta_4, \Delta_5, \Delta_6$ sextuplet 6*4

hhl: $\Delta_1 = \Delta_2, \Delta_3 = \Delta_4, \Delta_5 = \Delta_6$ triplet 3*4

$h0l$: $\Delta_1 = \Delta_6, \Delta_2 = \Delta_3, \Delta_4 = \Delta_5$ triplet 3*4

$00l$: $\Delta_1 = \Delta_2 = \Delta_3 = \Delta_4 = \Delta_5 = \Delta_6 = 0$ singlet 2

hexagonal > orthorhombic ($i = 3$)

hkl: $\Delta_1 = \Delta_4, \Delta_2 = \Delta_3, \Delta_5 = \Delta_6$ triplet 3*8

hhl: $\Delta_1 = \Delta_2, \Delta_5$ doublet 8+4

$h0l$: $\Delta_1 = \Delta_5, \Delta_2$ doublet 4+8

$00l$: $\Delta_1 = \Delta_2 = \Delta_5 = 0$ singlet 2.

1.4. Tetragonal to orthorhombic

Desymmetrization from tetragonal to orthorhombic implies a transformation index $i = 2$ and splitting into doublets is expected. The equations for peak (component) positions and displacements are

$$\frac{1}{d^2_{hkl_t}} = \frac{h^2}{a^2} + \frac{k^2}{a^2} + \frac{l^2}{c^2} \tag{32}$$

$$\frac{1}{d^2_{hkl_o}} = \frac{h^2}{a^2} + \frac{k^2}{b^2} + \frac{l^2}{c^2} = \frac{h^2}{a^2} + \frac{k^2}{a^2(1+\delta a/a)^2} + \frac{l^2}{c^2} \tag{33}$$

$$\frac{1}{d^2_{khl_o}} = \frac{k^2}{a^2} + \frac{h^2}{b^2} + \frac{l^2}{c^2} = \frac{k^2}{a^2} + \frac{h^2}{a^2(1+\delta a/a)^2} + \frac{l^2}{c^2} \tag{34}$$

$$\Delta_1 = (33) - (32) = \frac{k^2}{a^2}(\frac{1}{(1+\delta a/a)^2} - 1) \approx -\frac{k^2}{a^2}(2\frac{\delta a}{a}) \tag{35}$$

$$\Delta_2 = (34) - (32) = \frac{h^2}{a^2}(\frac{1}{(1+\delta a/a)^2} - 1) \approx -\frac{h^2}{a^2}(2\frac{\delta a}{a}) \tag{36}$$

and the split patterns for general and special reflections are shown in the following scheme and Fig. S4.

CONDITION: DISPLACEMENTS, SPLIT PATTERN

hkl: Δ_1, Δ_2 doublet 8+8

hhl:	$\Delta_1 = \Delta_2$	singlet 8
$hk0$:	Δ_1, Δ_2	doublet 4+4
$h0l$:	$\Delta_1 = 0, \Delta_2$	doublet 4+4
$hh0$:	$\Delta_1 = \Delta_2$	singlet 4
$h00$:	$\Delta_1 = 0, \Delta_2$	doublet 2+2
$00l$:	$\Delta_1 = \Delta_2 = 0$	singlet 2

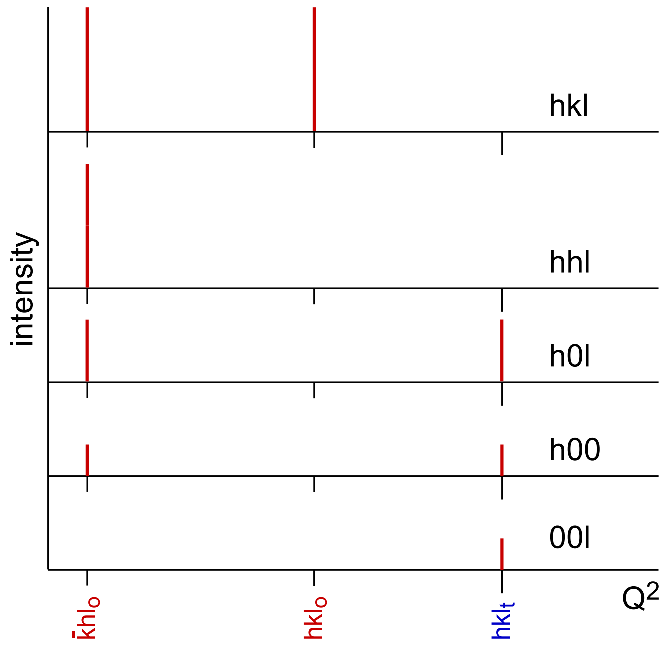

Fig. S4. Peak splitting of general hkl and special reflections after unit cell symmetry relaxation from tetragonal to orthorhombic. For special reflections, some displacements may become 0 or degenerate forming singlets. Actual displacements depend on the values of hkl (here $hkl = 321$), but the split pattern remains the same for a given class of reflections.

1.5. Orthorhombic to monoclinic

Similar to the preceding case, desymmetrization from orthorhombic to monoclinic implies a transformation index $i = 2$, now caused by allowing $\gamma = 90 + \delta\gamma$ (note that the "first" setting is used to warrant consistency with previous sections). The

equations for peak (component) positions and displacements are

$$\frac{1}{d^2_{hkl_o}} = \frac{h^2}{a^2} + \frac{k^2}{b^2} + \frac{l^2}{c^2} \tag{37}$$

$$\frac{1}{d^2_{hkl_m}} = [\frac{h^2}{a^2} + \frac{k^2}{b^2} - \frac{2hk}{ab}\cos\gamma]/\sin^2\gamma + \frac{l^2}{c^2} = \\ = \frac{h^2}{a^2}\frac{1}{S'} + \frac{k^2}{b^2}\frac{1}{S'} + \frac{hk}{ab}\frac{C'}{S'} + \frac{l^2}{c^2} \tag{38}$$

$$\frac{1}{d^2_{hkl_m}} = [\frac{h^2}{a^2} + \frac{k^2}{b^2} + \frac{2hk}{ab}\cos\gamma]/\sin^2\gamma + \frac{l^2}{c^2} = \\ = \frac{h^2}{a^2}\frac{1}{S'} + \frac{k^2}{b^2}\frac{1}{S'} - \frac{hk}{ab}\frac{C'}{S'} + \frac{l^2}{c^2} \tag{39}$$

$$\Delta_1 = (38) - (37) = \frac{h^2}{a^2}(\frac{1}{S'} - 1) + \frac{k^2}{b^2}(\frac{1}{S'} - 1) + \frac{hk}{ab}\frac{C'}{S'} = \\ = \frac{\sin\delta\gamma}{\cos^2\delta\gamma}[(\frac{h^2}{a^2} + \frac{k^2}{b^2})\sin\delta\gamma + \frac{2hk}{ab}] \approx \frac{hk}{ab}2\sin\delta\gamma \tag{40}$$

$$\Delta_2 = (39) - (37) = \frac{h^2}{a^2}(\frac{1}{S'} - 1) + \frac{k^2}{b^2}(\frac{1}{S'} - 1) - \frac{hk}{ab}\frac{C'}{S'} = \\ = \frac{\sin\delta\gamma}{\cos^2\delta\gamma}[(\frac{h^2}{a^2} + \frac{k^2}{b^2})\sin\delta\gamma - \frac{2hk}{ab}] \approx -\frac{hk}{ab}2\sin\delta\gamma \tag{41}$$

where C' and S' are defined as C and S in equations (21) and (22) but with $\gamma = (90 + \delta\gamma)^{\rm o}$ and $C' \to 0, S' \to 1$.

The split pattern is shown in the following scheme and Fig. S5. Similar to the case for desymmetrization from cubic to rhombohedral (section 1.2) a barycentre is observed, at $B_m = (h^2/a^2 + k^2/b^2)\sin\delta\gamma \approx 0$, which applies to all reflections except $00l$.

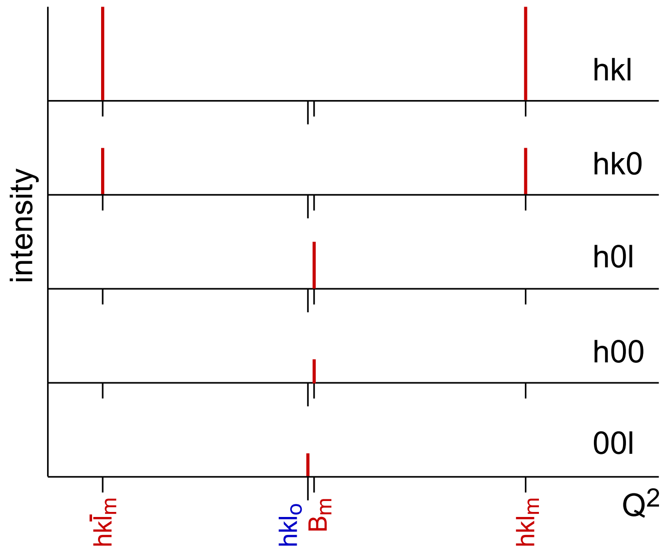

Fig. S5. Peak splitting of general hkl and special reflections after unit cell symmetry relaxation from orthorhombic to monoclinic. For special reflections, some displacements may become B_m or 0 and degenerate forming singlets. Actual displacements depend on the values of hkl (here $hkl = 321$), but the split pattern remains the same for a given class of reflections.

CONDITION: DISPLACEMENTS, SPLIT PATTERN

hkl:	Δ_1, Δ_2	doublet 4+4
$hk0$:	Δ_1, Δ_2	doublet 2+2
$h0l, 0kl$:	$\Delta_1 = \Delta_2 = B_m$	singlet 4
$h00, 0k0$:	$\Delta_1 = \Delta_2 = B_m$	singlet 2
$00l$:	$\Delta_1 = \Delta_2 = 0$	singlet 2

1.6. Monoclinic to anorthic (triclinic)

Desymmetrization from monoclinic to anorthic implies a transformation index $i = 2$, now caused by allowing $\alpha = 90 + \delta\alpha, \beta = 90 + \delta\beta$ (note that a setting with $\alpha \approx \beta \approx 90^{\circ}, \gamma \neq 90^{\circ}$ is used to warrant consistency with previous sections). The equations for

peak (component) positions and displacements are

$$\frac{1}{d^2_{hkl_m}} = [\frac{h^2}{a^2}\frac{W_a}{S^*} + \frac{k^2}{b^2}\frac{W_a}{S^*} + \frac{l^2}{c^2}W_a - \frac{2hk}{ab}\frac{W_a}{S^*}\cos\gamma]/W_a \tag{42}$$

$$\begin{aligned}\frac{1}{d^2_{hkl_a}} = [&\frac{h^2}{a^2}\cos^2\delta\alpha + \frac{k^2}{b^2}\cos^2\delta\beta + \frac{l^2}{c^2}S^* + \\ &+ \frac{2hk}{ab}(\sin\delta\alpha\sin\delta\beta - \cos\gamma) - \frac{2kl}{bc}(\sin\delta\beta\cos\gamma - \sin\delta\alpha) - \\ &- \frac{2lh}{ca}(\cos\gamma\sin\delta\alpha - \sin\delta\beta)]/W_a\end{aligned} \tag{43}$$

$$\begin{aligned}\frac{1}{d^2_{hk\bar{l}_a}} = [&\frac{h^2}{a^2}\cos^2\delta\alpha + \frac{k^2}{b^2}\cos^2\delta\beta + \frac{l^2}{c^2}S^* + \\ &+ \frac{2hk}{ab}(\sin\delta\alpha\sin\delta\beta - \cos\gamma) + \frac{2kl}{bc}(\sin\delta\beta\cos\gamma - \sin\delta\alpha) + \\ &+ \frac{2lh}{ca}(\cos\gamma\sin\delta\alpha - \sin\delta\beta)]/W_a\end{aligned} \tag{44}$$

$$\begin{aligned}\Delta_1 = (43) - (42) = [&\frac{h^2}{a^2}(\cos^2\delta\alpha - \frac{W_a}{S^*}) + \frac{k^2}{b^2}(\cos^2\delta\beta - \frac{W_a}{S^*}) + \\ &+ \frac{l^2}{c^2}(S^* - W_a) + \frac{2hk}{ab}(\sin\delta\alpha\sin\delta\beta - \cos\gamma + \frac{W_a}{S^*}\cos\gamma) - \\ &- \frac{2kl}{bc}(\sin\delta\beta\cos\gamma - \sin\delta\alpha) - \\ &- \frac{2lh}{ca}(\cos\gamma\sin\delta\alpha - \sin\delta\beta)]/W_a\end{aligned} \tag{45}$$

$$\begin{aligned}\Delta_2 = (44) - (42) = [&\frac{h^2}{a^2}(\cos^2\delta\alpha - \frac{W_a}{S^*}) + \frac{k^2}{b^2}(\cos^2\delta\beta - \frac{W_a}{S^*}) + \\ &+ \frac{l^2}{c^2}(S^* - W_a) + \frac{2hk}{ab}(\sin\delta\alpha\sin\delta\beta - \cos\gamma + \frac{W_a}{S^*}\cos\gamma) + \\ &+ \frac{2kl}{bc}(\sin\delta\beta\cos\gamma - \sin\delta\alpha) + \\ &+ \frac{2lh}{ca}(\cos\gamma\sin\delta\alpha - \sin\delta\beta)]/W_a\end{aligned} \tag{46}$$

where use has been made of equations (6) and (7) and $W_a = \sin^2\gamma - \sin^2\delta\alpha - \sin^2\delta\beta + 2\sin\delta\alpha\sin\delta\beta\cos\gamma \rightarrow \sin^2\gamma = S^*$.

The expressions (45) and (46) for the displacements from the monoclinic parent position cannot be approximated but the distance between the two split positions

$$\Delta_2 - \Delta_1 = (44) - (43) = \frac{4l}{cW_a}[\frac{k}{b}(\cos\gamma\sin\delta\beta - \sin\delta\alpha) + \\ + \frac{h}{a}(\cos\gamma\sin\delta\alpha - \sin\delta\beta)] \quad (47)$$

is fairly straightforward to calculate and may be useful to get the contribution of lattice symmetry relaxation to peak width.
